# Developmental and Epileptic Encephalopathy 76: Case Report and Review of Literature

**DOI:** 10.3390/children9121967

**Published:** 2022-12-15

**Authors:** Xiaodi Han, Jie Deng, Chunhong Chen, Xiaohui Wang, Fang Fang, Hua Li, Jie Luo, Jie Wu

**Affiliations:** 1 Department of Neurology, Beijing Children’s Hospital, Capital Medical University, National Center for Children’s Health, Beijing 100045, China; 2 Neonatal Center, Beijing Children’s Hospital, Capital Medical University, National Center for Children’s Health, Beijing 100045, China; 3 Department of Emergency, Beijing Children’s Hospital, Capital Medical University, National Center for Children’s Health, Beijing 100045, China

**Keywords:** early-onset epileptic encephalopathy, developmental and epileptic encephalopathy type 76, ACTL6B, genetic testing

## Abstract

Previous studies have suggested that the ACTL6B monoallelic variant is responsible for an autosomal dominant inherited intellectual developmental disorder with severe speech and ambulation deficits. The clinical phenotype of developmental and epileptic encephalopathy type 76 (DEE76) due to ACTL6B biallelic variants was first reported in 2019, with an autosomal recessive mode of inheritance. In this paper, we report on a child in China with DEE76 caused by a compound heterozygous variant of the ACTL6B gene, and we review the literature on ACTL6B gene variants causing DEE76 with complete clinical information. Including our case 1, the genotype and phenotypic characteristics of 18 children from 14 families are summarized. All 18 cases are autosomal recessive, including 12 with homozygous variants and six with compound heterozygous variants. A total of 17 variants have been reported so far, including 14 variants of the loss function. We summarize the clinical features using Human Phenotype Ontology (HPO) terms. We find that DEE76, caused by the ACTL6B biallelic variant, is an early-onset drug-refractory epilepsy with global developmental delay^HP:0001263^, hypertonia^HP:0001276^, and microcephaly^HP:0000252^, and imaging is characterized by brain delayed myelination^HP:0012448^. Our case of DEE76 had not been reported when the patient underwent genetic testing in 2018, and the diagnosis was clarified by the reanalysis of the data 2 years later, being the first reported Chinese patient and the only one in which the application of a ketogenic diet for antiepileptic treatment may have been effective.

## 1. Introduction

Early-onset epileptic encephalopathies (EOEE) is a general term for a group of epileptic encephalopathies with onset within 6 months of life [1], whose frequent seizures and epileptic discharges lead to progressive neurological function damage. Affected children commonly present with drug-resistant epilepsy and generalized developmental delay. In recent years, it has been discovered that the etiology of around 70% of EOEE cases is related to hereditary factors [2,3]. At present, it is believed that the developmental disorders of children with EOEE are not only caused by frequent seizures and epileptiform discharges, but are more directly affected by genetic variation, so developmental and epileptic encephalopathies (DEE) have been proposed. This term highlights the important role of the genetic background in the clinical phenotype; even when seizures are controlled, the developmental delay due to genetic etiology is difficult to resolve [4].

In this study, we describe a boy who had the ACTL6B compound heterozygous variant, and the final diagnosis was DEE 76 (DEE76, OMIM: 618468). We summarize the clinical features using HPO terms [5]. Additionally, the literature on ACTL6B gene variation causing DEE76 with complete clinical information was summarized, to improve clinicians’ understanding of DEE76 and promote the diagnosis and treatment of the disease.

## 2. Case Report

### 2.1. Clinical Manifestation

The child that we reported was the first child of a normal couple without close relatives. She was delivered by cesarean section at 40 weeks and 4 days of gestation due to uterine inertia and an occipital transversal position. Her birth weight was 3750 g, and there was no history of asphyxia, hypoglycemia, or pathologic jaundice during the perinatal period. Family history was negative. One hour after birth, she had seizures^HP:0001250^ without obvious causes, which were presented as head tilted back, upper left eyes staring, hands clenching, both upper limbs flexing and shaking, treadmill movements of both lower limbs, and purple around the mouth, which lasted around 10 s and spontaneously relieved, with a frequency of 5–6 times a day. On the 18th day after birth, the seizure was the same as before. After the seizure, she was accompanied by crying and was successively treated with phenobarbital, vitamin B6, levetiracetam, midazolam, and oxcarbazepine. She was given a ketogenic diet in addition to antiepileptic medication at the age of 4 months. The seizures were gradually controlled by the age of 6 months. She was treated with ACTH at 9 months of age, and her seizures decreased slightly. After this, she was successively treated with oxazepine, sodium valproate, topiramate, nitrazepam, and lacosamine, and continued her ketogenic diet. Her seizures were gradually controlled after one and a half years, but her global developmental delay^HP:0001263^ was prominent and she showed hypertonia^HP:0001276^. After 1 year, the child had no obvious seizures and was treated with sodium valproate, oxcarbazepine, lacosamine, and nitrazepam combined with a ketogenic diet, with a ketogenic ratio of 2.5:1 and blood ketone of around 2.5 mmol/L. Oral baclofen reduced muscle tension and we insisted on rehabilitation training, but developmental progress was limited. At the age of 2 years and 10 months, the child underwent outpatient follow-up examination: she could raise her head, laugh, and babble. She could not chase after animals, sit alone, or speak. She regularly had involuntary movements such as head tilting back and hand and foot twisting. The head circumference was 45 cm, the forehead was narrow, the eye distance was wide, the nose was round, the lips were thick, the muscle strength of the limbs was level IV, the muscle tone was increased, and the pathological signs were negative. A few days later, the child developed pneumonia and respiratory failure after catching a cold, followed by seizures. She was admitted to the emergency intensive care unit of our hospital, where she received endotracheal intubation ventilator-assisted ventilation and active anti-infection. Three days later, she died of central respiratory failure.

### 2.2. Neuroimaging and VEEG 

**Figure 1:**
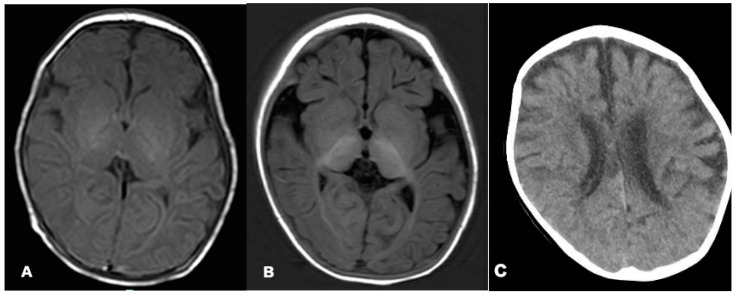
Brain MRI of the patient at the ages of 1 month (**A**) and 6 months (**B**). Brain CT of the patient at the age of 2 years and 10 months (**C**). Axial T1-weighted images suggested decreased periventricular white matter on both sides (**A**,**B**). Brain CT showed atrophic changes in both cerebral hemispheres (**C**).

On the 27th day after birth, magnetic resonance imaging (MRI) of the brain revealed a blurred T1 high signal in the hindlimb of the internal capsule, a lack of white matter, and a high T2 signal. Brain MRI at 6 months revealed deep sulci, delayed myelination^HP:0012448^, little white matter, and thin corpus callosum. Brain CT at 2 years and 10 months demonstrated brain atrophy^HP:0012444^ (Figure 1). Video electroencephalogram (VEEG) at 1 month and 15 days showed interictal multifocal epileptiform discharges, noticeable bilaterally in the occipital region. Frequent clinical seizures of focal origin and electrical seizures (discharges that can travel from one hemisphere to the opposite side during a seizure) were identified. At 8 months, VEEG revealed extensive, multifocal epileptic discharges between seizures, and spastic–tonic seizures were observed. At 1 year and 2 months, VEEG was monitored for slow background activity, with no occipital-dominated rhythm, interictal multifocal epileptiform discharges, and asymmetric tonic seizures originating in the back of the head. At 1 year and 6 months, VEEG displayed no occipital dominant rhythm, bilateral occipital spikes, and slow waves, and no seizures were detected.

### 2.3. Genetic Analysis

**Figure 2:**
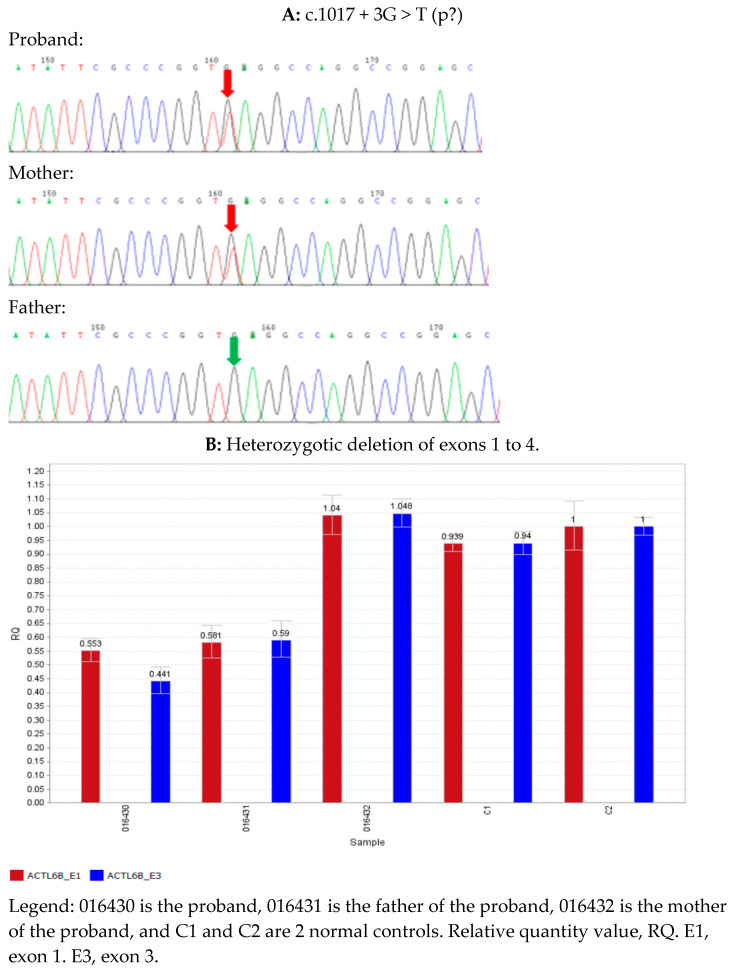
**Summary of ACTL6B variants.** (**A**) Sanger sequencing confirmed that the proband and its mother had ACTL6B c.1017 + 3G > T heterozygous variant, while the father was a wild type. (**B**) Quantitative polymerase chain reaction (qPCR) results: The QR values of the proband and its parents, ACTL6B gene E1, were 0.553, 0.581, and 1.04, respectively. The QR values of the proband and its parents, ACTL6B gene E3, were 0.441, 0.59, and 1.048, respectively, indicating that the proband and its father had a heterozygous deletion in the above region, and the proband’s mother had a normal copy number in this region. Generally, QR < 0.1 was considered as a homozygous deletion, 0.3 < QR < 0.7 as a heterozygous deletion, 0.7 < QR < 1.3 as normal, and 1.3 < QR < 1.8 as a haploid duplication.

The patient underwent single whole-exome sequencing, copy number variation, and mitochondrial gene testing at 5 months, and no pathogenic variants that were extremely consistent with the phenotype were found. However, combined with the clinical presentation and auxiliary examination results, the possibility of a hereditary etiology was still considered. At 2 years and 5 months, the trio of sequencing was completed, and we used the whole-exon next-generation sequencing (NGS) data analysis method to analyze the deletion duplication of all exons (more than 190,000 exons) in the whole exon group [6]. The genomic DNA was extracted from the peripheral blood of the proband and its parents. Then, we applied a hybrid capture approach to acquire the DNA library after the genomic DNA was fragmented, ligated, amplified, and propagated. Next, a high-throughput sequencing platform (Illumina, San Diego, CA, USA) was employed to detect the human whole-exome regions of 20,099 genes or nuclear genes associated with mitochondrial diseases and their flanking intron regions, to obtain possible pathogenic variants. The sequencing data were aligned to UCSC hg19 with BWA software. Variants were filtered and annotated through ANNOVAR software. We further validated the selected variants of the proband by Sanger sequencing or quantitative polymerase chain reaction. Furthermore, we evaluated the pathogenicity of the variants with reference to the 2015 American College of Medical Genetics and Genomics (ACMG) guidelines [7]. We found that the proband had a compound heterozygous variant of the ACTL6B gene (transcript number: NM_016188.4) (Figure 2A,B). One of these was chr7/hg19, chr7:g.100252621_100254104del, namely the ACTL6B gene exon 1–4 heterozygous deletion, from the father, and this variant has not been reported in the literature. It is a loss-of-function variant (PVS1); the frequency in the normal database is extremely low (PM2), the associated disease DEE76 and the proband’s clinical phenotype match (PP4), and the variant is defined as a pathogenic variant (PVS1 + PM2 + PP4). The remaining one, c.1017 + 3G > T—that is, the 1017 + 3 base in the intron region—was mutated from G to T, and it was from the mother. This variation has not been reported in the literature and has not been detected in normal human databases (PM2); bioinformatics analysis software MaxEntScan, NNSPLICE, and GeneSplicer all predicted that the variant might affect the normal shearing of mRNA (PP3). Pathogenic variants were detected in trans positions in recessive genetic diseases (PM3), and the associated disease DEE76 was consistent with the clinical phenotype of the proband (PP4), which was defined as a suspected pathogenic variant (PM2 + PP3 + PM3 + PP4).

## 3. Literature Review

With “ACTL6B, developmental epileptic encephalopathy” as the keyword, no relevant reports were retrieved in the Wanfang database or the CNKI database (the database was established until November 2022). Using “ACTL6B, Developmental and Epileptic Encephalopathies” as the search term, the PubMed database (the database was established until November 2022) was searched, and five English literature studies were retrieved, reporting a total of 17 cases of ACTL6B-related DEE (DEE76) [8,9,10]. Including our case 1, the genotype and phenotypic characteristics of 18 children from 14 families were summarized (Table 1). All 18 cases were autosomal recessive, including 12 cases of homozygous variants and six cases of compound heterozygous variants. A total of 17 variantal sites were found. There were eight nonsense variants, three missense variants, three deletion variants, two splice variants, and one frameshift variant. Among the 18 patients, 13 were female and 5 were male. The onset age of epilepsy ranged from newborn to 3 years old, of which 92.3% (12/13) had onset within 1 year. Among the seizure types, tonic seizures accounted for 70% (7/10), epileptic spasm seizures accounted for 40% (4/10), and myoclonic seizures accounted for 30% (3/10). The various types of seizures can occur simultaneously or sequentially. Major signs other than epilepsy included hypertonia^HP:0001276^ (18/18), axial hypotonia HP:0008936 (15/18), feeding difficulties^HP:0011968^ (9/18), and dysphagia^HP:0002015^ (3/18). There were 12 cases of microcephaly^HP:0000252^. All 18 patients had global developmental delay^HP:0001263^, and 12 of them were severe. Of these, 16 had a full EEG examination, of which nine had multifocal epileptic discharges, seven showed slowing of background activity, and four had generalized epileptic discharges during the interval. Brain MRI results were provided in 16 cases, of which 10 showed delayed myelination^HP:0012448^ with or without reduced brain volume, thin corpus callosum^HP:0033725^, and brain atrophy^HP:0012444^.

To summarize, the prognosis of 18 children from 14 families was considered (including one case in this group). The median age of 18 patients at last assessment was 4.25 years (range: 5 months to 10 years). Four of them died (including one in this group). The median age at death was 3.5 years (range: 2 years to 5 years). The final developmental assessment was provided in 15 patients, all of whom had developmental delays, and in 10 of whom there was a clear indication that the child was unable to talk or walk. Of the 17 reported cases, three were treated with 3–9 antiepileptics, two with the ketogenic diet, and one with ACTH, with no reduction in seizures. The child that we are reporting on received seven antiepileptic drugs in succession, including phenobarbital, levetiracetam, ocazepine, sodium valproate, topiramate, nitrazepam, and lacosamine. At 4 months, when antiepileptic drugs were found to be ineffective, the ketogenic diet was introduced. At 9 months, ACTH was applied for 2 weeks and the ketogenic diet continued. At the last follow-up assessment, the child was able to hold her head up, laugh, and babble. However, she was not able to sit alone or talk, and occasionally had involuntary movements such as tilting her head back and twisting her hands and feet. Unfortunately, the child died at the age of 2 years and 10 months from central respiratory failure following an infection.

## 4. Discussion

The ACTL6B gene, located at 7q22.1, encodes a subunit of the neuron-specific chromatin remodeling complex nBAF, which is crucial for neuronal differentiation, dendritic extension, synaptic function, and long-term memory [11,12]. ACTL6B knockout mice showed increased perinatal mortality, impaired dendritic growth in the nervous system, and defective axonal myelin development, suggesting that the ACTL6B allele variant may affect dendritic growth and axonal development in postmortem neurons [13]. The ACTL6B gene has two inheritance modes: autosomal dominant and autosomal recessive. Among them, autosomal dominant inheritance leads to mental retardation and severe language and walking impairment (Intellectual Developmental Disorder with Severe Speech and Ambulation Defects, IDDSSAD, OMIM: 618470). The clinical phenotypes of 10 children reported so far include intellectual disability, walking impairment, severe language impairment, hypotonia, Rett-like stereotypes, and mild facial deformities (wide mouth, diaphragm, bulbous nose) [8]; this phenotype has no seizures. Autosomal recessive inheritance leads to DEE76, which is characterized by drug-resistant epilepsy, severe global developmental delay^HP:0001263^, hypertonia^HP:0001276^, microcephaly^HP:0000252^, and brain delayed myelination^HP:0012448^, with early postnatal onset [8,9,10], a phenotype that was included in the OMIM in June 2019. In this case, seizures were gradually brought under control after 1 year and 6 months, but the global developmental delay^HP:0001263^ and hypertonia^HP:0001276^ were prominent. We also noted severe global developmental delay^HP:0001263^ at 2 years and 6 months old, microcephaly^HP:0000252^, slightly abnormal facial features (narrow forehead, wide eye distance, round nose, thick lips), and hypertonia^HP:0001276^. In this case, the patient had an early onset age, a transition in seizure type and EEG presentation, and prominent mental retardation, so the diagnosis of EOEE was clear. In terms of etiology, there was insufficient evidence for metabolic and structural factors, so genetic factors were more likely to be considered. The final genetic data re-analysis found the ACTL6B compound heterozygous pathogenic variant, and the phenotype of this child was in excellent agreement with DEE76 caused by the ACTL6B allelic variant, thus establishing a diagnosis of DEE76, the first reported in China. The MRI of a child with DEE76 showed diffuse delayed myelination^HP:0012448^ [8,9,10] and the child also had prominent myelodysplasia of the brain, which may be a characteristic imaging manifestation of the disease. The ACTL6B gene is thought to be involved in neurodevelopment and dendritic growth after mitosis, thus affecting synaptic development and myelination. Increased muscle tone and spasticity in children is thought to be caused by myelination of the back of the brain.

Dominant and recessive variants in the ACTL6B gene cause the disease via different molecular pathways. Bell [8] demonstrated that the ACTL6B biallelic variant mimics the loss-of-function and gain-of-function of monoallelic variant in a human cellular context. The 18 cases of ACTL6B biallelic variant-related DEE76 have been found to involve a total of 17 variant sites; with loss-of-function variants (nonsense variant, frameshift variant, classical splicing variant, start codon variant, multiple exon deletions), a total of 14 were found. The 10 cases of monoallelic variant-related IDDSSAD reported so far involve a total of two variant sites, all of which are de novo missense variants, of which nine cases are c.1027G > A (p.Gly343Arg), and one case is c.230A > G (p.Asp77Gly) [8]. The ACTL6B biallelic variant has not been reported in the pathogenic family of monoallelic variants, and it has been suggested that it may be a loss-of-function variant that does not fit the pathogenic mechanism of gain-of-function in monoallelic variants.

At the onset of the disease, the child was actively improving as a person with full exome sequencing and copy number variation testing, but no cause was found. At that time (2018), DEE76 with biallelic variation of the ACTL6B gene had not been reported. Nevertheless, based on the clinical characteristics of this patient, it is still highly regarded as a genetic etiology. After a lapse of 2 years, the data were re-analyzed based on the family’s complete full-exome sequencing, and the latest database was queried to verify the child’s diagnosis. In 2014, Wright [14] et al. performed whole-exome sequencing on 1133 children with severe developmental disabilities and their parents, with 27% being diagnosed. A re-analysis of full-exome sequencing data in 2017 showed that the diagnosis rate for full-exome sequencing increased from 27% to 40%. With the rapid advances in genomics, through family-wide exome sequencing and genome-wide sequencing, continued improvements in clinical phenotypes, the optimization of algorithms, the updating of databases, and the re-analysis of previous genetic data can significantly enhance the positive rate of genetic diagnosis, help guide therapy, improve prognosis, and provide genetic counseling. The etiology of epilepsy can be divided into six categories: hereditary, structural, metabolic, immune, infectious, and unknown etiology [4]. Around 30% of epilepsy cases can be explained by a hereditary etiology, especially in EOEE. In recent years, with the improvement in the level of genetic diagnosis, the genetic etiology and pathophysiological mechanism of epilepsy have been more deeply understood. In the past, the pathogenic gene detection rate of EOEE was less than 10%. With the application of targeted next-generation sequencing, causative genes have been identified in more than 25% of EOEE cases [15], suggesting that genetic factors play an important role in the pathogenesis of EOEE. DEE also emphasizes the decisive influence of the genetic background in the clinical phenotype. Even if epileptic seizures caused by genetic factors can be improved or even controlled, the developmental delay is still prominent. The in-depth exploration of the genetic etiology of EOEE has important and far-reaching significance for its pathogenesis, genotyping, individualized treatment, prognosis, and genetic counseling.

## 5. Conclusions

In conclusion, the possibility of DEE76 should be considered in children with drug-resistant epilepsy with early onset after birth, accompanied by global developmental delay^HP:0001263^, hypertonia^HP:0001276^, microcephaly^HP:0000252^, and delayed myelination^HP:0012448^. For children with DEE, care should be taken to check for hereditary causes, and those considering hereditary causes should complete relevant genetic tests in a timely manner. Even if the test results are negative, if hereditary causes are still considered based on clinical characteristics, treatment outcomes, and prognosis follow-up, it is necessary to continuously re-analyze gene sequencing data and search and compare them with updated databases to improve the positive rate of genetic diagnosis, thereby guiding treatment and improving prognosis.

## Figures and Tables

**Table 1:** Phenotypic features of 18 patients with ACTL6B recessive variants.

Patient	Total (N = 18)
Gender	F 72% (13/18)
	M 28% (5/18)
Epilepsy	
Onset age of seizures	<1 month 28% (5/18)
	1 month~1 year 39% (7/18)
	>1 year 5% (1/18)
	NR 28% (5/18)
Seizure type	Tonic seizures 39% (7/18)
	Myoclonic seizures 22% (4/18)
	Epileptic spasms 17% (3/18)
	NR 44% (8/18)
Initial EEG	Multifocal epileptic activity 50% (9/18)
	Generalized slowing of background rhythms 39% (7/18)
	Generalized epileptiform discharges 22% (4/18)
	NR 11% (2/18)
Major signs besides epilepsy	Developmental delay 100% (18/18)
	Hypertonia 100% (18/18)
	Axial hypotonia 83% (15/18)
	feeding difficulties 50% (9/18)
	dysphagia 17% (3/18)
Dysmorphic features	Microcephaly 67% (12/18)
	Facial abnormalities 33% (6/18)
	NR 11% (2/18)
	Normal 5% (1/18)
MRI	Hypoplasia of corpus callosum 56% (10/18)
	Delayed myelination 56% (10/18)
	Cerebral and cerebellar atrophy 33% (6/18)
	NR 11% (2/18)
	Normal 5% (1/18)

Legend: F, female. M, male. NR, not reported.

## Data Availability

The datasets used and/or analyzed during the current study are available from the corresponding author upon reasonable request.
